# Phylogeny and the inference of evolutionary trajectories

**DOI:** 10.1093/jxb/eru118

**Published:** 2014-04-22

**Authors:** Lillian Hancock, Erika J. Edwards

**Affiliations:** Department of Ecology and Evolutionary Biology, Brown University, Box G-W, 80 Waterman St, Providence, RI 02912, USA

**Keywords:** C4 photosynthesis, crassulacean acid metabolism, evolution, intermediate phenotypes, phylogenetic approach, phylogeny.

## Abstract

Most important organismal adaptations are not actually single traits, but complex trait syndromes that are evolutionarily integrated into a single emergent phenotype. Two alternative photosynthetic pathways, C_4_ photosynthesis and crassulacean acid metabolism (CAM), are primary plant adaptations of this sort, each requiring multiple biochemical and anatomical modifications. Phylogenetic methods are a promising approach for teasing apart the order of character acquisition during the evolution of complex traits, and the phylogenetic placement of intermediate phenotypes as sister taxa to fully optimized syndromes has been taken as good evidence of an ‘ordered’ evolutionary trajectory. But how much power does the phylogenetic approach have to detect ordered evolution? This study simulated ordered and unordered character evolution across a diverse set of phylogenetic trees to understand how tree size, models of evolution, and sampling efforts influence the ability to detect an evolutionary trajectory. The simulations show that small trees (15 taxa) do not contain enough information to correctly infer either an ordered or unordered trajectory, although inference improves as tree size and sampling increases. However, even when working with a 1000-taxon tree, the possibility of inferring the incorrect evolutionary model (type I/type II error) remains. Caution is needed when interpreting the phylogenetic placement of intermediate phenotypes, especially in small lineages. Such phylogenetic patterns can provide a line of evidence for the existence of a particular evolutionary trajectory, but they should be coupled with other types of data to infer the stepwise evolution of a complex character trait.

## Summary

We simulated ordered and unordered character evolution across phylogenetic trees to understand how tree size, models of evolution, and sampling efforts influence the ability to detect an evolutionary trajectory.

## Introduction

One of the most compelling and enduring problems in evolution is the origin and assembly of complex character traits or syndromes. How does natural selection gradually modify genes and phenotypes to assemble new and fully integrated suites of characters? How can these serial modifications or ‘steps’ along an evolutionary trajectory be reconstructed? Crassulacean acid metabolism (CAM) and C_4_ photosynthesis in plants are two key examples of such integrated trait syndromes. Evolving numerous times in distantly related lineages, these morphologically and physiologically distinct adaptive syndromes use an internal plant carbon-concentrating mechanism that improves the efficiency of C_3_ photosynthesis under conditions of high heat, drought, and/or low atmospheric CO_2_ (Edwards and Ogburn, 2012). The genetic and morphological underpinnings of C_4_ photosynthesis are especially well studied, and importantly, many ‘intermediate’ phenotypes have been discovered and analysed (Kennedy and Laetsch, 1974; Powell, 1978; Hattersley, 1986; Monson and Moore, 1989; Kellogg, 1999; Besnard *et al.*, 2009; Feodorova *et al.*, 2010; Christin *et al.*, 2011; Sage *et al.*, 2012). These C_3_–C_4_ intermediates have been essential in the construction of a theoretical C_4_ evolutionary trajectory, which maps out the transitions from C_3_ to C_4_ metabolism as a series of ordered events, with anatomical changes preceding most of the biochemical changes, and a precursor (a photorespiratory pump carbon-concentrating mechanism) preceding the activation of PEP Caryboxylase and the C_4_ cycle (Sage, 2004, 2012). When C_3_, intermediate, and C_4_ phenotypes are mapped across phylogenies that span the C_3_ to C_4_ spectrum, the intermediates are often placed as sister to a fully C_4_ group, and the intermediate state is then interpreted as the ancestral condition of their shared node (i.e. Molluginaceae, Neurachninae, and *Flaveria*) (McKown *et al.*, 2005; Christin *et al*., 2011, 2012). The further interpretation is that, to evolve a full C_4_ syndrome, a lineage must first pass through this particular intermediate condition.

Similar to C_4_ photosynthesis, ‘intermediate’ CAM-like phenotypes have been discovered and analysed (Sternberg *et al*., 1983, 1984; Ting, 1985; Harris and Martin, 1991; Guralnick and Jackson, 2001; Winter *et al.*, 2008; Silvera *et al.*, 2010). These CAM-like phenotypes fall along a C_3_ to CAM spectrum and are often difficult to characterize as they are variably expressed and appear indistinguishable from C_3_ species under non-stressful conditions (Ting, 1985; Cushman, 2001; Dodd *et al.*, 2002; Lüttge, 2004). Nevertheless, hypotheses posit that these CAM intermediates (CAM cycling and facultative CAM) may act as evolutionary steps between a typical C_3_ plant and a fully expressed CAM syndrome (Monson, 1989; Guralnick and Jackson, 2001; Sage, 2002; Edwards and Ogburn, 2012), although their phylogenetic distribution with respect to C_3_ and CAM lineages is less clear.

**Fig. 1.**
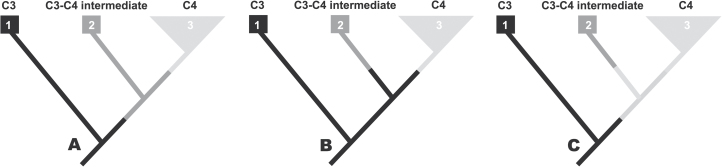
Interpreting the evolution of intermediate phenotypes along the C_4_ evolutionary trajectory. (A) The intermediate phenotype (C_3_–C_4_ intermediate) evolved once, prior to the divergence of lineages 2 and 3, and so the extant intermediate represents the ancestral condition of lineage 3, the C_4_ clade. (B) The C_3_–C_4_ intermediate and the C_4_ phenotype each evolved once after the divergence of lineages 2 and 3, and so the extant intermediate does not represent the ancestral condition of lineage 3 (C_4_ clade) rather the C_3_ phenotype represents the ancestral condition of both lineages 2 and 3. (C) The C_4_ phenotype evolved prior to the divergence of lineages 2 and 3 and the C_3_–C_4_ intermediate evolved after the divergence of lineage 2; in this scenario, the C_4_ phenotype represents the ancestral condition of the C_3_–C_4_ intermediate. Each of these scenarios only requires two evolutionary steps, though (A) is the most common interpretation in the literature.

There are likely to be a limited set of functional intermediate phenotypes that are frequently passed through during the C_3_ to C_4_ transition, and the same is likely to be true during CAM evolution as well. This study addresses not whether a trajectory (or a set of trajectories) exists, but rather how useful is the phylogenetic approach for uncovering them? The situation is more complicated than it appears at first glance (Fig. 1), because all of the information on intermediate states are found in currently living species—taxa that are not ancestral to any other taxa. This study uses the distribution of traits among the living ‘tips’ of phylogenies to infer the tempo and mode of character evolution and to reconstruct past evolutionary events; however, in small trees with few character transitions, there is little information to work with. For example, in the common scenario pictured in Fig. 1, it is typically inferred that the intermediate phenotype evolved once, prior to the divergence of the C_3_–C_4_ and C_4_ lineages, and so the extant C_3_–C_4_ intermediate represents the ancestral condition of the C_4_ clade. (e.g. Fig. 1A). That is certainly a plausible scenario, but no more plausible than the alternatives in Fig. 1B and 1C: all scenarios result in only two evolutionary transitions, but only that in Fig. 1A supports the hypothesized ordered trajectory of C_4_ evolution. In general, a phylogenetic approach would need a richer ‘tree-trait space’ (i.e. many more branches, and many more character transitions) to discern the relative likelihood of these three alternatives. While the three taxon case in Fig. 1 is an especially information-poor scenario, unfortunately, many empirical datasets do not really contain many more transitions, as intermediate phenotypes are generally rare in comparison to the numbers of species with fully evolved syndromes and also require significant effort to detect (McKown *et al.*, 2005; Marshall *et al.*, 2007; Vogan *et al.*, 2007; Christin *et al*., 2011, 2012; Ocampo *et al.*, 2013).

This study explored the power of the phylogenetic approach to identify ordered and unordered evolutionary trajectories by simulating character evolution under different evolutionary scenarios and across trees of various sizes and shapes. Because complete taxon sampling of a focal clade is very rare in phylogenetic studies, this study also investigated how limited taxon sampling across a tree influences the evolutionary signal of these ordered and unordered data sets. The findings suggest that the taxonomic scale of many phylogenetic studies is inadequate for inferring ordered evolution, and surprisingly, increasing tree size and the number of evolutionary transitions has a relatively modest positive effect on the accuracy of model inference. On the positive side, limited taxon sampling does not seem to greatly reduce the overall evolutionary signal of the data sets.

## Materials and methods

### Simulating character evolution

To simulate ordered, discrete character data, eight unique Q matrices were built representing four character states along an evolutionary trajectory: state 1 → state 2 → state 3 → state 4. A Q matrix is an instantaneous transition rate matrix (Tables 1 and 2) where each cell within the matrix corresponds to the probability of transitioning between two states.

**Table 1.** Ordered Q matricesExamples of Q matrices (equal rates) used to simulate an ordered evolutionary trajectory. Character evolution was modelled using a continuous-time Markov model, where each transition between character states is assigned an instantaneous rate.

	State 1	State 2	State 3	State 4
Reversible Q matrix				
State 1	–1	1	0	0
State 2	1	–1	1	0
State 3	0	1	–1	1
State 4	0	0	1	–1
Non-reversible Q matrix				
State 1	–1	1	0	0
State 2	0	–1	1	0
State 3	0	0	–1	1
State 4	0	0	0	0

**Table 2.** Unordered Q matricesExamples of Q matrices (equal rates) used to simulate an unordered evolutionary trajectory. Character evolution was modelled using a continuous-time Markov model, where each transition between character states is assigned an instantaneous rate.

	State 1	State 2	State 3	State 4
Reversible Q matrix				
State 1	–3	1	1	1
State 2	1	–3	1	1
State 3	1	1	–3	1
State 4	1	1	1	–3
Non-reversible Q matrix				
State 1	–3	1	1	1
State 2	0	–2	1	1
State 3	0	0	–1	1
State 4	0	0	0	0

To specify order within the matrix, an instantaneous transition rate of ‘zero’ was assigned to all matrix cells that represent a ‘skip’ across states (e.g. 1 → 3, 2 → 4), whereas possible transitions were assigned integers 1, 2, or 3; integers correspond to relative, but not absolute, transition rates. Since all rows must sum to zero, the diagonal cells (e.g. 1→1, 2→2) within the matrix are equal to the sum of the off-diagonal elements within the rows (i.e. the diagonal cells can be negative so that rows sum to zero). Eight matrices were built to simulate different models of character evolution (i.e. with all transitions occurring at equal rates, with all rates different, with forward and reverse transitions between two characters at equal rates, and with high transition rates out of intermediate character states) (Supplementary Table S1 available at *JXB* online). Integers 1–3 were placed in matrix cells to reflect these four models of evolution. Four of the eight matrices were ordered reversible while the remaining matrices were not reversible (meaning that a transition from state 1 to state 2 was allowed, but a transition from state 2 to state 1 was not allowed) (Table 1). All analyses were done using the R Project for Statistical Computing.

This work also simulated unordered trait evolution using the same four character states with the goal of understanding the probability of incorrectly inferring an ordered trajectory. Q matrices were constructed to imply unordered evolution between each of the four character states, 1, 2, 3, and 4 (Table 2; Supplementary Table S2). In other words, transitions between states 1 to 3, 1 to 4, and 2 to 4 (impossible under the ordered model) were allowed to happen. All matrix cells were assigned integers 1, 2, or 3 unless the matrix was non-reversible (four of the eight simulating matrices).

**Fig. 2.**
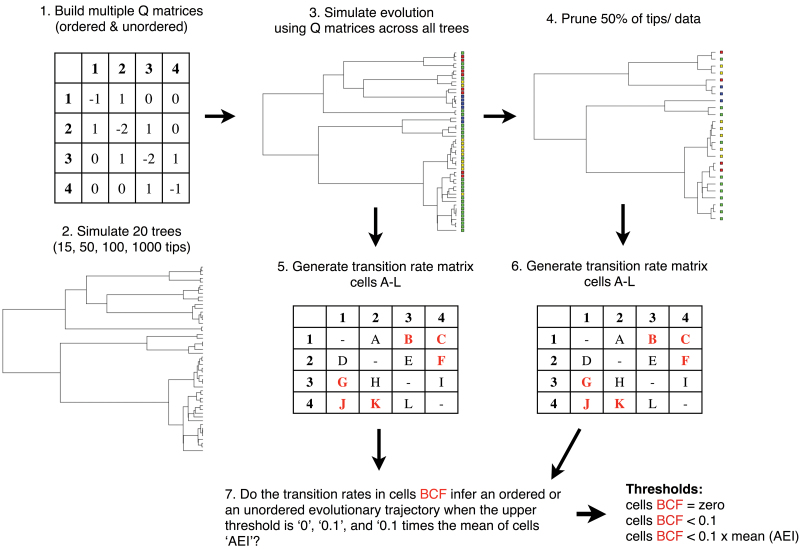
Schematic representation of methods used to simulate and analyse ordered and unordered character data (this figure is available in colour at *JXB* online).

Using the rtree function in the APE package (Paradis *et al.*, 2004), 20 unique ultrametric, coalescent 15-taxon, 50-taxon, 100-taxon, and 1000-taxon trees were randomly generated for the ordered and the unordered character simulations (Fig. 2). Trees generated with the coalescent generally capture a smaller fraction of tree shape than do birth–death models (Mooers *et al.*, 2007). With the sim.char function in the Geiger package (Harmon *et al.*, 2008), character evolution was simulated across each tree using the constructed matrices (Fig 2). For a given Q matrix and tree, character data was independently simulated 200 times, and the first 10 simulations containing all four states at the tree tips (1, 2, 3, 4) were selected for downstream analyses. More data was simulated than used because all four character states were not always present across extant tips in each simulation.

### Sensitivity analysis

To test for the effect of poor sampling on the ability to infer character evolution, a second, ‘pruned’ dataset was generated where 50% of the taxa (tips) and corresponding data were randomly removed from each of the original simulated data sets across ordered and unordered 50-taxon, 100-taxon, and 1000-taxon trees (Fig. 2). Fifty per cent of the taxa were not removed from the 15-taxon data set as this tree was already quite small.

Approximately 1200 ordered and unordered data simulations were generated for each of the 15-taxon, 50-taxon, 100-taxon, and 1000-taxon groups. This number is lower than the expected 1600 (20 trees × 8 matrices × 10 simulations of each matrix) because simulated data was removed (1) when all four character states were not realized among the 10 simulations when 50% of the data/tips were removed and (2) when the transition rates between states were too infinitely low (but not zero) to calculate (non-linear minimization error). When these errors ensued, simulations were removed from the data sets so the analysis could continue to run.

### Inferring character transition rates of simulated data

The next step used the simulated data as if a typical phylogenetic analysis was being started, with only a phylogeny and character data at the tips and no a priori knowledge of the underlying model of character evolution. Using the ace function in the package APE (Paradis *et al.*, 2004), the maximum likelihood estimates of each transition rate within the four-by-four matrix were calculated under a model that allowed each rate to vary independently (Fig. 2). Since the diagonal cells within the matrix are the sum of the off-diagonal elements within the rows, 12 transition rates (A–L) corresponding to the remaining matrix cells were saved to relevant tables (Table 3), which could then be compared to expected rates according to the generating matrix.

**Table 3.** Ancestral reconstruction transition rate matrixLetters A–L refer to the transition rates generated for each ancestral character state reconstruction. Transitions BCF and GJK are not possible under an ordered model; all transitions are possible under an unordered model.

	State 1	State 2	State 3	State 4
State 1	–	A	B	C
State 2	D	–	E	F
State 3	G	H	–	I
State 4	J	K	L	–

### Data analysis

The primary goal was to evaluate how well phylogenetic methods can detect an evolutionary trajectory using only the phylogenetic relationships and trait distributions of living taxa. Analyses were focused on comparing the forward transition rates among character states (cells A, B, C, E, F, and I in Table 3). Matrix cells B (transition of state 1 → state 3), C (state 1 → state 4), and F (state 2 → state 4) represent the transition rates that were expected to be zero when simulated under an ordered model (Table 1). For each simulation, this study evaluated how close to zero these rates were, using multiple thresholds for designating an estimated rate as essentially ‘zero’. In the most conservative threshold, B, C, and F cells of the estimated matrix all had to be zero for the inferred evolutionary model to be considered ‘ordered’. Using a more liberal threshold, all B, C, and F cells had to be lower than 0.1; and in the most liberal threshold, all B, C, and F cells had to have rates that were at least one magnitude lower than the average transition rate of cells A, E, and I. Then each simulated dataset was designated to be either an inferred ‘ordered’ or an ‘unordered’ evolutionary model, according to each threshold, and compared this to the actual model used to simulate the data.

## Results

**Fig. 3.**
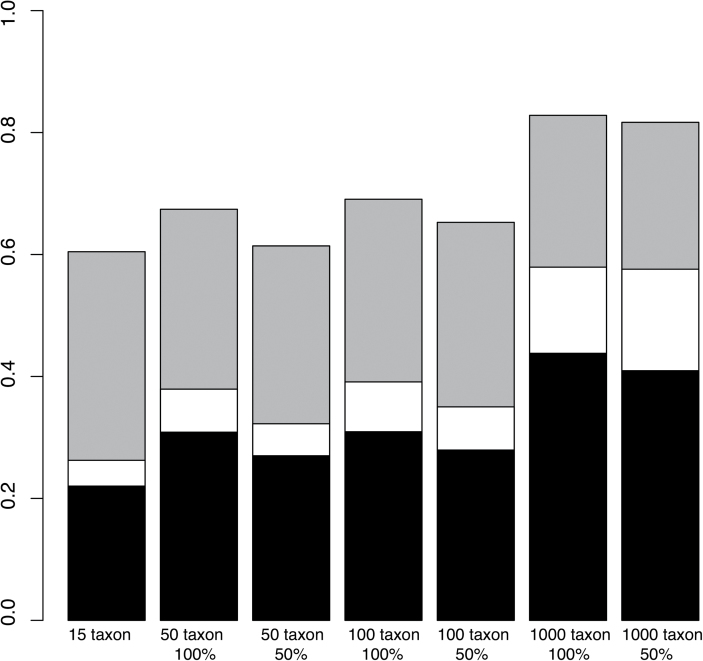
Probability of accurately inferring an ordered trajectory when the generating data is ordered for each taxon group at the three predefined thresholds, in order of decreasing stringency: (1) cells B, C, and F must be 0 (black); (2) cells B, C, and F must be less than 0.1 (white); (3) cells B, C, and F have rates at least one magnitude less than the average transition rate of cells A, E, and I (grey). The probability of inferring the correct evolutionary model increases with 1) taxon sample size and 2) loosening threshold stringency.

Using the most conservative threshold (all ‘zero’ cells must have inferred transition rates to be zero), it was exceedingly difficult to infer an ordered evolutionary trajectory, with only 22% of the 15-taxon tree simulations accepting an ordered model, which increased to only 44% in a 1000-taxon tree (Fig. 3). Relaxing the threshold of rates to be larger than but close to zero greatly improved the ability to infer the generating model: using a threshold where the ‘zero’ cells must be inferred to be at least one order of magnitude lower than the average rate of the ‘non-zero’ cells, many more of the ordered simulations could be classified as ordered, up to 60% in the 15-taxon case and 83% in the 1000-taxon case.

**Fig. 4.**
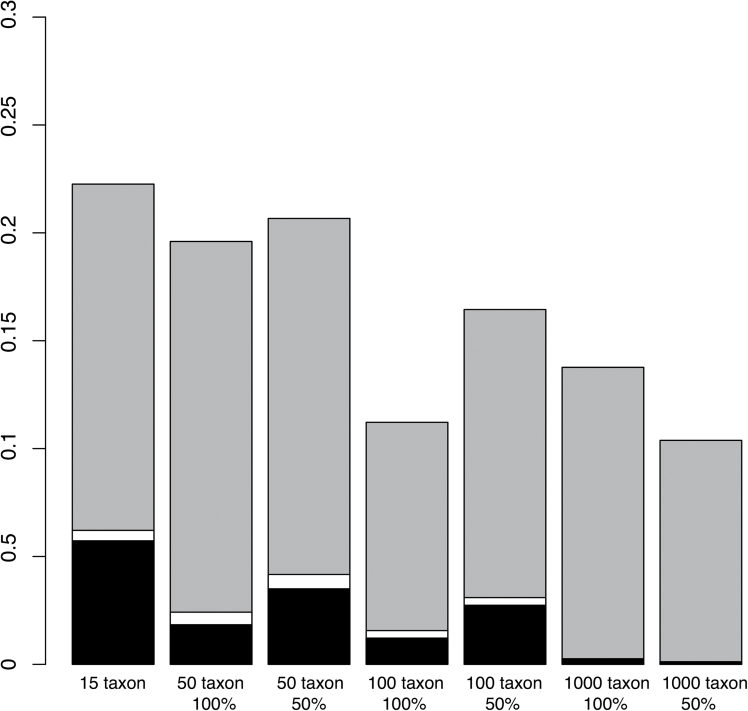
Probability of inferring an ordered evolutionary trajectory when the generating data is unordered (type I error) for each taxon group at the three predefined thresholds, in order of decreasing stringency: (1) cells B, C, and F must be 0 (black); (2) cells B, C, and F must be less than 0.1 (white); (3) cells B, C, and F have rates at least one magnitude less than the average transition rate of cells A, E, and I (grey). The probability of inferring a type I error decreases with 1) increasing taxon sample size and 2) increasing stringency.

However, relaxing the threshold had the additional complicating effect of also drastically increasing the frequency of assigning an ‘ordered’ model to simulations that were actually generated using an unordered matrix (type I error) (Fig. 4). The conservative threshold produced negligible type I error, with the exception of the 15-taxon tree (5.7% acceptance of an ordered model when the underlying data were unordered). However, relaxing the threshold consistently increased the propensity to label unordered data as ordered, from 14% in 1000-taxon trees to an alarming 22% in 15-taxon trees.

Increasing tree size consistently reduced the probability of both type I and type II errors, but this improvement was surprisingly modest. The 656% increase in tree size between the 15-taxon and the 1000-taxon groups resulted in only a 20% gain in inference accuracy. In a similar vein, reducing taxon sampling produced its predicted effect of reducing accuracy, but the magnitude of the effect was small, typically only reducing the number of correctly inferred models by several percentage points (Figs 3 and 4). The effect of missing data was larger in smaller datasets: in the 50-taxon dataset, simulating missing data resulted in an average 12% decrease in accuracy across the three thresholds, but this reduction was only 3% in the 1000-taxon dataset.

**Fig. 5.**
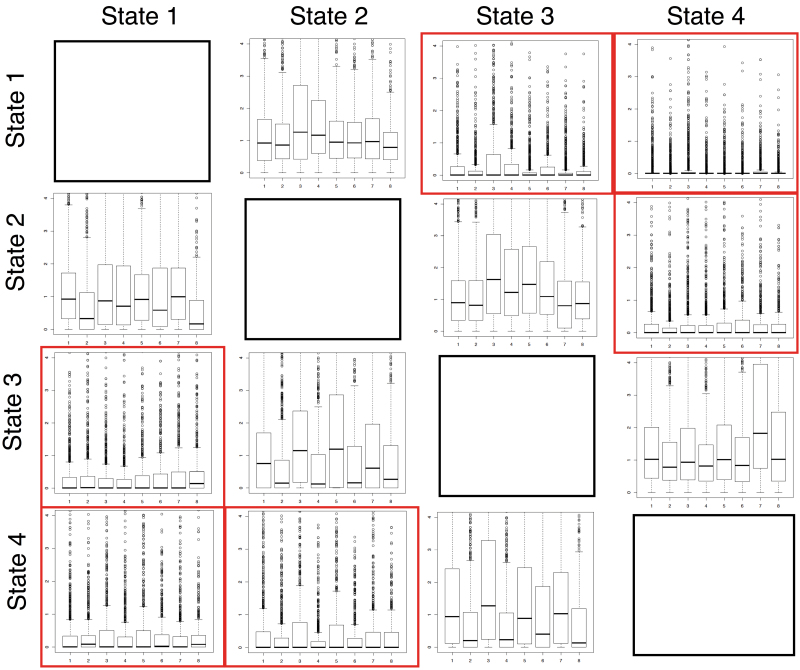
Four-by-four Q matrix represents the transition rates between character states 1–4 for the fully sampled 1000-taxon ordered data set. Box plots within each cell represent the generated transition rates for each of the eight simulating Q matrices. Forward and reverse ‘zero cells’ (not allowed to evolve under an ordered model) are outlined. Transition rates are consistently lower within the ‘zero’ cells and especially between state 1 and state 4 (this figure is available in colour at *JXB* online).

This study also looked at the individual transition rates generated from each of the eight Q matrices across all tree size classes separately, to determine whether data simulated under particular models of evolution (inferred from the given Q matrices; Supplementary Table S1 available at *JXB* online) were more indicative of an ordered evolutionary trajectory. No general patterns were detected across all matrices, and, consistent with the threshold analyses, transition rates were generally much higher in the ‘evolving’ cells, while the cells not allowed to evolve had rates close to or at zero (Fig. 5). By looking at the individual rates, this study was able to detect a consistent difference among the ‘zero’ cells: cell C, which represents a transition from state 1 to state 4, thereby skipping all intermediate character states, had much lower transition rates across all matrices than the other ‘zero’ cells (transitions 1 → 3 or 2 → 4). This pattern was observed consistently across all trees by matrix combinations.

## Discussion

Often, the distribution of intermediate character states across the tips of a phylogeny is used to infer the stepwise evolution of integrated trait syndromes, such as C_4_ photosynthesis. This study asked: how much power does the phylogenetic approach really have to detect ordered trait evolution? By simulating ordered and unordered character evolution across a diverse set of phylogenetic trees, this study investigated how tree size, model of evolution, and sampling efforts influence the ability of standard phylogenetic comparative tools to detect an evolutionary trajectory.

The findings highlight some significant limitations to this approach. Under the most data-poor scenario (a 15-taxon tree), the methods of inference were, at best, 60% effective at detecting ordered evolution, but this came with the serious cost of also incorrectly inferring order over 20% of the time. Increasing clade size improved the situation, but not as much as one might hope. Two results are somewhat uplifting. First, increasing clade size from 15 to 50 tips provided nearly the same benefit as increasing to 100 tips, suggesting that the biggest returns relative to investment might be at the small end of the spectrum. Second, in very large clades (1000 taxa), the effect of missing data appears to be quite negligible. This is especially good news for studies of CAM evolution, as many CAM-evolving clades are overwhelmingly speciose (e.g. orchids, bromeliads) and exhaustive taxon sampling is, at this stage, simply not feasible. While this is heartening, it comes with the caveat that the missing data were simulated randomly with respect to character state. In both C_4_ and CAM syndromes, much more is likely to be known about the distribution of fully evolved pathways, as they are easily detectable with stable carbon isotopes (Bender, 1971; Bender *et al.*, 1973; Sternberg *et al.*, 1984; O’Leary, 1988; Farquhar *et al.*, 1989). The sampling in empirical studies may therefore be biased towards including known C_4_ and CAM plants at the exclusion of other (potentially still unidentified) intermediates, and this particular pattern of sampling bias was not addressed in the current study.

Admittedly, there is more than one way to infer an ordered trajectory based on phylogenetic patterns, and these analyses are fairly simplistic. In future work it might be useful to compare various phylogenetic approaches. An alternative to the estimated rates approach used here might be an actual tally of inferred transitions, using stochastic mapping (Bollback, 2006; for implementation in grasses see Roalson, 2011) or parsimony reconstruction. Another might be to simply compare the fit of a suite of character evolution models and choose the best-fit model with likelihood scores. This alternative approach was tested on a data subset using the Geiger module fitDiscrete in R (Harmon *et al.*, 2008). Preliminary analyses indicate that the rate matrix approach may work better with smaller trees, and the model-fitting approach increases in accuracy more quickly as trees become larger. At the same time (and possibly related), a model-fitting approach appears more sensitive to poor taxon sampling. While these different methods clearly need further examination, one benefit to the current approach is that it allows for a closer look at all the possible transitions and thus allows for a better intuition of where the methods are working and where they are not. For instance, matrix cell C (Table 3), which represents a transition from state 1 → state 4, had the lowest transition rate across all simulations (Fig. 3). This is an important detail, as it suggests that a type II error is likely caused by incorrectly inferring transitions into and out of intermediate states. Thus, in the four character state scenario tested, the methods successfully identified that intermediate states were passed through from the initial first state to the final fourth state; however, these methods were not successful in identifying the exact order of the intermediate states.

It is important to realize that the phylogenetic placement of intermediate states is an essential component of inferring evolutionary trajectories; this work has simply illustrated that phylogenetic inference, like anything else, is not infallible and should not be given any sort of primary importance when evaluating different scenarios supported by different kinds of evidence (Christin *et al.*, 2010). A key example of this ‘priority’ type of treatment is the recent study by Ocampo *et al.* (2013), who found a C_3_–C_4_ intermediate lineage nested within the C_4_ species *Portulaca*. In spite of acknowledging the anatomical and biochemical differences among the C_4_ lineages that surround this intermediate, the authors seemed to eventually be arguing that, because ancestral state reconstruction supported a reversion from C_4_ to a C_3_–C_4_ intermediate, this is the order of trait evolution that should be accepted. The current simulation study suggests that the number of character transitions in *Portulaca* are too few to provide the information necessary to feel confident in a phylogenetic reconstruction. And indeed, as evidence grows about this particular example of C_4_ evolution, it seems increasingly clear that there have been multiple parallel realizations of C_4_ in different *Portulaca* clades (Christin *et al.*, 2014).

Two recent studies (Heckmann *et al.*, 2013; Williams *et al.*, 2013) have used alternative approaches to tease apart the evolutionary assembly of C_4_ photosynthesis. Heckmann *et al.* (2013) inferred the relative fitness gain of each biochemical change along the C_4_ trajectory, using modelled photosynthetic rate as a measure of fitness, to create the ‘adaptive landscape’ of C_4_ evolution. They found that the intermediate C_3_–C_4_ phenotypes are indeed transitory states, as the relative fitness gains associated with the realization of these intermediate phenotypes were minor compared to the fitness gain of a fully optimized C_4_ syndrome. Williams *et al.* (2013) took an entirely different approach and, using empirical data from 43 studies, characterized 16 biochemical, anatomical, and cellular characteristics associated with C_4_ photosynthesis and C_3_–C_4_ intermediates to build a transition network connecting C_3_ and C_4_ photosynthesis. Then, using a Bayesian approach, they sampled the biologically relevant ‘paths’ through the network to infer the most common order in which phenotypic changes have occurred along the C_4_ trajectory. Findings from this study suggest that the evolutionary trajectory of C_4_ photosynthesis is somewhat ordered but that trait acquisition along the trajectory is flexible.

Both of these studies were explicitly non-phylogenetic, and the incorporation of phylogenetic information, especially in the approach of Williams *et al.* (2013) is an exciting opportunity for improvement. Unquestionably, phylogeny has a central role in the study of both C_4_ and CAM evolution: it identifies new lineages for research, can provide a timeline for transitions, and identifies ecological and organismal correlates. As phylogenetic biologists, the authors truly think that phylogeny can and should be integrated into all areas of biological research. It is simply important to understand the caveats and limitations inherent in inferring past evolutionary events with only a handful of data from currently living species. A phylogenetic approach provides valuable evidence for a particular evolutionary trajectory, but should be considered for what it is—an informed hypothesis that can be supported or refuted with additional data.

## Supplementary material

Supplementary data are available at *JXB* online.

Supplementary Table S1. Ordered Q matrices.

Supplementary Table S2. Unordered Q matrices.

Supplementary Data
