# Supplementary material for: Phylogeny and the inference of evolutionary trajectories
Source: J Exp Bot. 2014 Apr 22;65(13):3491–8. doi: 10.1093/jxb/eru118 (PMC4085962; doi:10.1093/jxb/eru118)
Supplement: Supplementary Data [file supp_65_13_3491__index.html]

Phylogeny and the inference of evolutionary trajectories — Phylogeny and the inference of evolutionary trajectories — Supplementary Data 

# Phylogeny and the inference of evolutionary trajectories

## Supplementary Data

Data files

**Files in this Data Supplement:**

- Supplementary Data - Supplementary Data
