# Supplementary material for: Phylogeny and the inference of evolutionary trajectories
Source: J Exp Bot. 2014 Apr 22;65(13):3491–8. doi: 10.1093/jxb/eru118 (PMC4085962; doi:10.1093/jxb/eru118)
Supplement: Supplementary Data [file supp_eru118_Supplemental_tables_final.pdf]

Supplemental Table S1. Ordered Q matrices.

**Matrix 1**	State 1	State 2	State 3	State 4
State 1	-1	1	0	0
State 2	1	-2	1	0
State 3	0	1	-2	1
State 4	0	0	1	-1

**Matrix 2**	State 1	State 2	State 3	State 4
State 1	-1	1	0	0
State 2	0	-1	1	0
State 3	0	0	-1	1
State 4	0	0	0	0

**Matrix 3**	State 1	State 2	State 3	State 4
State 1	-2	2	0	0
State 2	1	-4	3	0
State 3	0	2	-3	1
State 4	0	0	3	-3

**Matrix 4**	State 1	State 2	State 3	State 4
State 1	-2	2	0	0
State 2	0	-3	3	0
State 3	0	0	-1	1
State 4	0	0	0	0

**Matrix 5**	State 1	State 2	State 3	State 4
State 1	-1	1	0	0
State 2	1	-3	2	0
State 3	0	2	-3	1
State 4	0	0	1	-1

**Matrix 6**	State 1	State 2	State 3	State 4
State 1	-1	1	0	0
State 2	0	-2	2	0
State 3	0	0	-1	1
State 4	0	0	0	0

**Matrix 7**	State 1	State 2	State 3	State 4
State 1	-1	1	0	0
State 2	1	-2	1	0
State 3	0	1	-4	3
State 4	0	0	1	-1

**Matrix 8**	State 1	State 2	State 3	State 4
State 1	-1	1	0	0
State 2	0	-1	1	0
State 3	0	0	-3	3
State 4	0	0	0	0

Supplemental Table S2. Unordered Q matrices

**Matrix 1**	State 1	State 2	State 3	State 4
State 1	-3	1	1	1
State 2	1	-3	1	1
State 3	1	1	-3	1
State 4	1	1	1	-3

**Matrix 2**	State 1	State 2	State 3	State 4
State 1	-3	1	1	1
State 2	0	-2	1	1
State 3	0	0	-1	1
State 4	0	0	0	0

**Matrix 3**	State 1	State 2	State 3	State 4
State 1	-5	2	1	2
State 2	1	-5	3	1
State 3	2	2	-7	3
State 4	1	1	3	-4

**Matrix 4**	State 1	State 2	State 3	State 4
State 1	-5	2	1	2
State 2	0	-4	3	1
State 3	0	0	-3	3
State 4	0	0	0	0

**Matrix 5**	State 1	State 2	State 3	State 4
State 1	-6	1	2	3
State 2	1	-5	2	2
State 3	2	2	-5	1
State 4	3	2	1	-6

**Matrix 6**	State 1	State 2	State 3	State 4
State 1	-6	1	2	3
State 2	0	-4	2	2
State 3	0	0	-1	1
State 4	0	0	0	0

**Matrix 7**	State 1	State 2	State 3	State 4
State 1	-5	1	1	3
State 2	1	-5	1	3
State 3	1	1	-5	3
State 4	1	1	1	-3

**Matrix 8**	State 1	State 2	State 3	State 4
State 1	-5	1	1	3
State 2	0	-4	1	3
State 3	0	0	-3	3
State 4	0	0	0	0
